# Supplementary material for: Dynamical evolution of CO2 and H2O on garnet electrolyte elucidated by ambient pressure X-ray spectroscopies
Source: Nat Commun. 2024 Mar 30;15:2777. doi: 10.1038/s41467-024-47071-4 (PMC10981710; doi:10.1038/s41467-024-47071-4)
Supplement: Supplementary file 1 — Supplementary Information [file 41467_2024_47071_MOESM1_ESM.pdf]

Supplementary Information for

Dynamical Evolution of CO_2 and H_2O on Garnet Electrolyte Elucidated by Ambient Pressure X-ray Spectroscopies

Nian Zhang,[1,2†] Guoxi Ren,[2†] Lili Li,[3†] Zhi Wang,[4] Pengfei Yu,[2] Xiaobao Li,[2] Jing Zhou,[3] Hui Zhang,[1,2]* Linjuan Zhang,[3] Zhi Liu,[5*] and Xiaosong Liu[2,5,6]*,

[1] Shanghai Synchrotron Radiation Facility, Shanghai Advanced Research Institute, Chinese Academy of Sciences, Shanghai 201204, China

[2] State Key Laboratory of Functional Materials for Informatics, Shanghai Institute of Microsystem and Information Technology, Chinese Academy of Sciences, Shanghai 200050, China

[3] Shanghai Institute of Applied Physics, Chinese Academy of Sciences, Shanghai 201204, China

[4] Institute of Semiconductors, Chinese Academy of Sciences, Beijing 100089, China

[5] School of Physical Science and Technology, Shanghai Tech University, Shanghai 201210, China

[6] National Synchrotron Radiation Laboratory, University of Science and Technology of China, Hefei, 230026, China

[†]These authors contributed equally: Nian Zhang, Guoxi Ren, Lili Li

*Corresponding author: Hui Zhang, Zhi Liu, Xiaosong Liu

E-mail addresses: huizhang@mail.sim.ac.cn (Hui Zhang), liuzhi@shanghaitech.edu.cn (Zhi Liu), xsliu19@ustc.edu.cn (Xiaosong Liu).

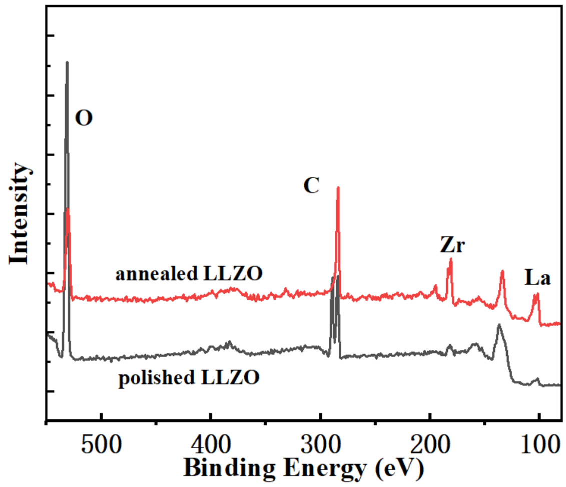

Supplementary Fig. 1 | Comparison of XPS survey on LLZO surface before and after annealing at room temperature. After vacuum annealing and cooling to room temperature, the signals of Li_2CO_3 almost disappear, La and Zr on the surface of LLZO are significantly enhanced.

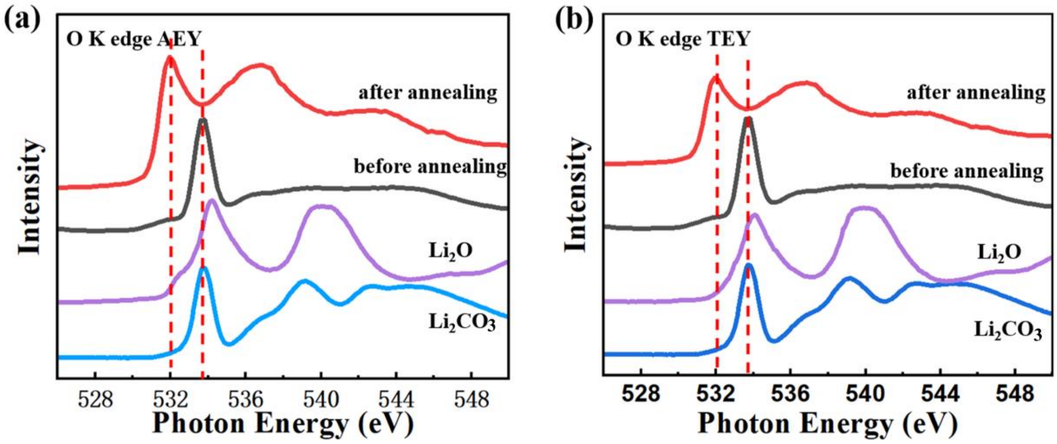

Supplementary Fig. 2 | Comparison of AEY and TEY spectra on LLZO surface before and after annealing at room temperature. a O K edge AEY and **b** TEY spectra of LLZO before and after vacuum annealing at room temperature. Li_2O and Li_2CO_3 are listed as reference samples. A small signal of LLZO can be found at ~531.9 eV in both AEY and TEY spectrum before annealing, indicating that the thickness of the contamination layer is several nanometers. After vacuum annealing at room temperature, Li_2CO_3 is hardly visible in the AEY and TEY spectra, indicating that very little Li_2CO_3 is generated on the surface during the cooling process.

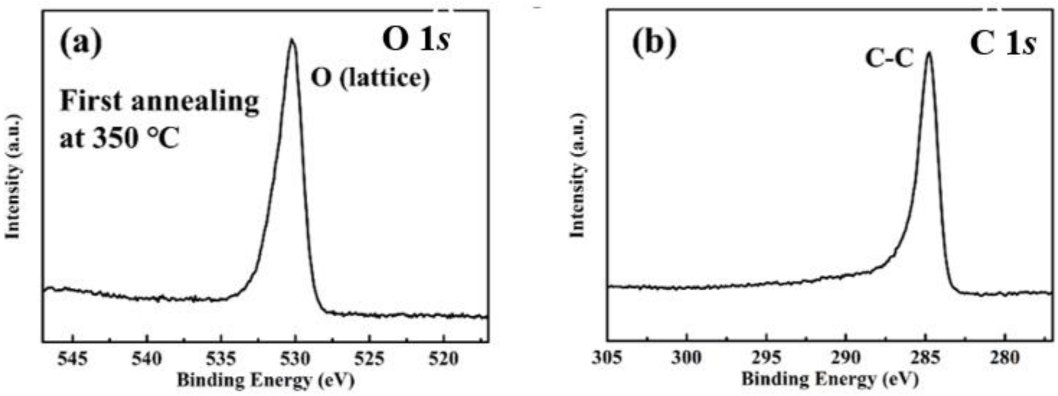

Supplementary Fig. 3 | XPS spectra of LLZO surface when first vacuum annealing at 350 ℃. a O 1*s* and **b** C 1*s* XPS spectra of LLZO when first vacuum annealing at 350 ℃ corresponding to process b in Fig. 2 of the main text. The results indicate clean LLZO surface at high temperature with only contaminated C can be achieved by vacuum annealing.

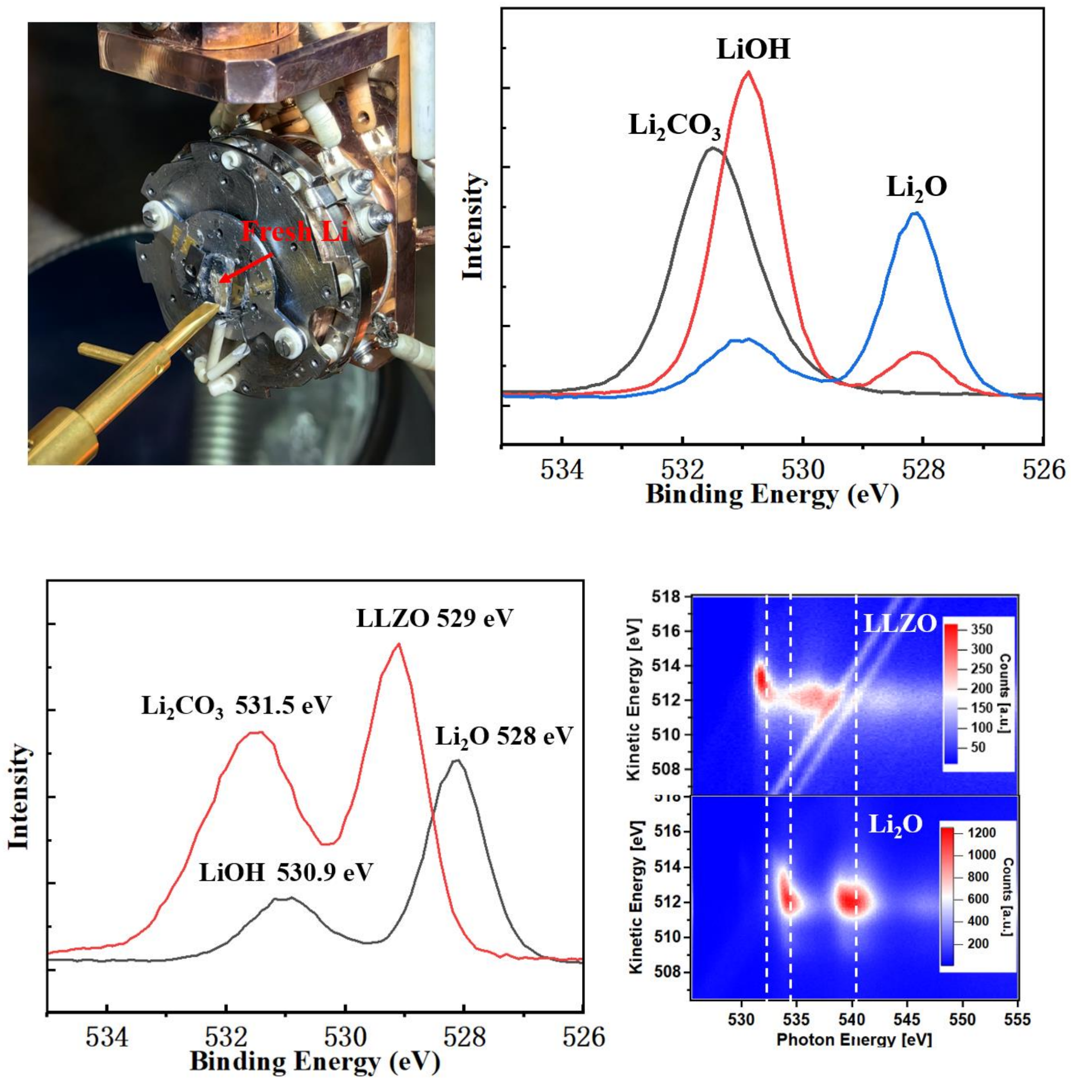

Supplementary Fig. 4 | Obtaining pure standard spectra of lithium containing species through *in situ* treatment of metallic lithium and excluding the presence of Li_2O on the surface of annealed LLZO through mRAS. Li metal was *in situ* scraped using a Wobble-Stick with sharp blade. By utilizing near ambient pressure technology, we can obtain pure Li_2O, LiOH and Li_2CO_3. Through these XPS data of reference samples, we can fix Li_2O peak position at 528 eV with a FWHM of 1.35 eV; LiOH at 530.9 eV with a FWHM of 1.61 eV, Li_2CO_3 at 531.5 eV with a FWHM of 1.75 eV. These parameters make the peak fitting in our article more accurate. The mRAS comparison of annealed LLZO and Li_2O excludes the generation of Li_2O during the annealing process.

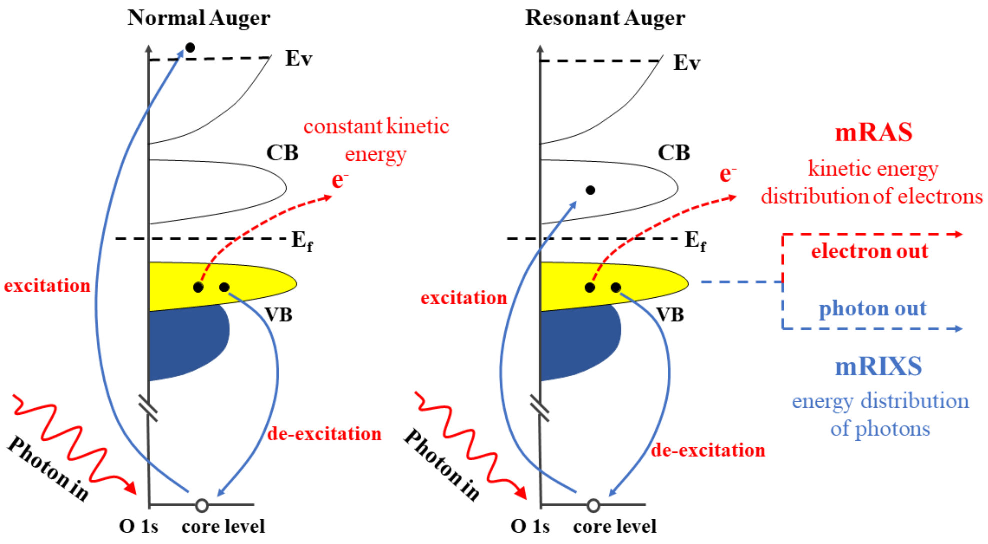

Supplementary Fig. 5 | Schematic diagram of the fundamental process of normal Augur and resonant Auger. Compared to normal Auger, resonance Auger contains valence band information of the sample. The core level electron can be excited under irradiation, followed by two parallel de-excitation channels (photon-in-electron-out vs. photon-in-photon-out), where the energy distribution of emitted electrons and photons can be further resolved into mapping of resonant Auger spectroscopy (mRAS) and mapping of resonant inelastic X-ray scattering (mRIXS). The two-dimensional maps provide energy resolution along both incident photon energy and emitted electron/photon axis, which can well disentangle the spectra overlapping effect. For soft X-ray, the photon yield is one percent of the electron yield and the efficiency of electronic energy analyzers is often much stronger than that of grating spectrometers, which means that mRAS has much lower requirements for photon flux and acquisition time than mRIXS. Using light from a bending magnet beamline, we can also complete a mRAS mapping in steps of 0.1 eV in 15 minutes. In addition, the mRAS and AEY method can be implemented at any synchrotron radiation XPS end-station with almost no need to add new hardware.

	Area of O Li_2CO_3 (O 1*s*)	Area of C Li_2CO_3 (C 1*s*)	O atom/C atom
**UHV after 1 mbar CO_2**	58397.4	5981.4	3.75
**1 mbar CO_2**	5120.6	1709.1	1.15
**0.5 mbar CO_2**	17056.3	3317.9	1.98
**1×10^{-2} mbar CO_2**	56698.7	5536.9	3.94
**1×10^{-4} mbar CO_2**	45065	4385	3.95
**1×10^{-6} mbar CO_2**	31251	3085	3.88
**after O_2 annealing**	26203	2240.8	4.49
**vacuum annealing**	23912	2035	4.54
**Pure Li_2CO_3**	381281	59000	2.48

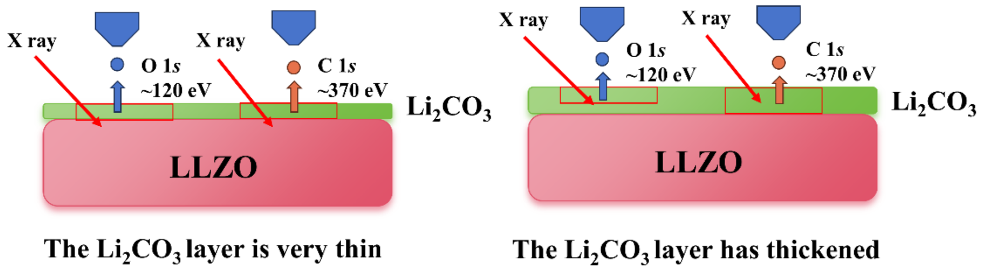

Supplementary Fig. 6 | Atomic ratio of O/C for Li_2CO_3 calculated through O 1*s* and C 1*s* during CO_2 reaction. For CO_2 reaction, in the table, we calculated the atomic ratio of O/C using the photoionization cross-sections of O (0.3383) and C (0.1308) at 650 eV. The O/C is maintained at a stable value ~3.9 during the CO_2 pressure range 10^{-6}-10^{-2} mbar. The stability of the ratio means that within this pressure range, the surface is relatively pure Li_2CO_3 without LiOH and the layer is very thin, and the X-ray can detect all information within the layer. Compared with pure Li_2CO_3 ~2.48, the ratio is higher. This phenomenon can be explained simply by the figure.

When 650 eV X-ray is incident on the surface of the material, the photoelectron kinetic energy of O is about 120 eV, while that of C is 370 eV. The detected depth of C is larger, so for pure Li_2CO_3, the proportion of C will be higher. For the results above 0.5 mbar CO_2, O/C is affected by the gas peak, the higher the CO_2 pressure, the lower the ratio. Interestingly, after we pumped 1 mbar CO_2 back to the vacuum, the O/C ratio slightly decrease to 3.75, indicating that the thickness of the Li_2CO_3 layer slightly exceeded the detection depth of O photoelectrons.

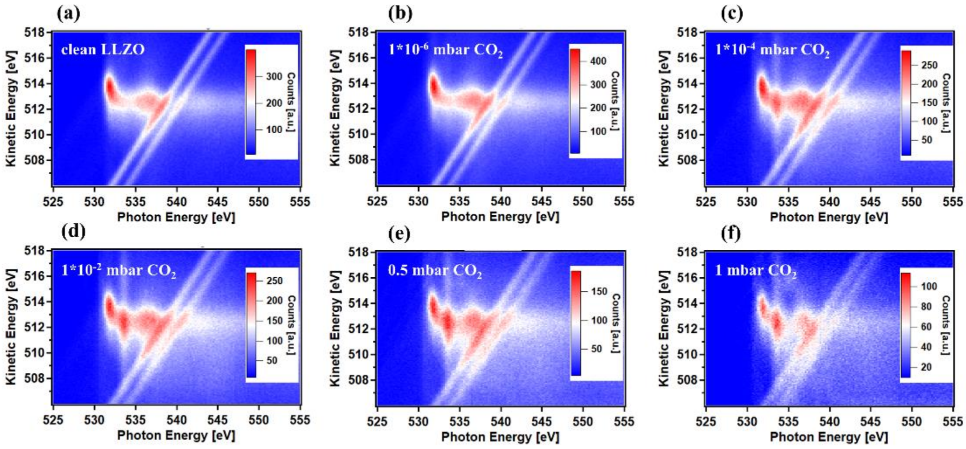

Supplementary Fig. 7 | The AP-mRAS spectra of LLZO surface during the introduction of CO_2. Clear intensity increasing of Li_2CO_3 can be observed from UHV to 1x10^{-2} mbar CO_2. The ratio of LLZO/ Li_2CO_3 becomes stable when the pressure over 1x10^{-2} mbar.

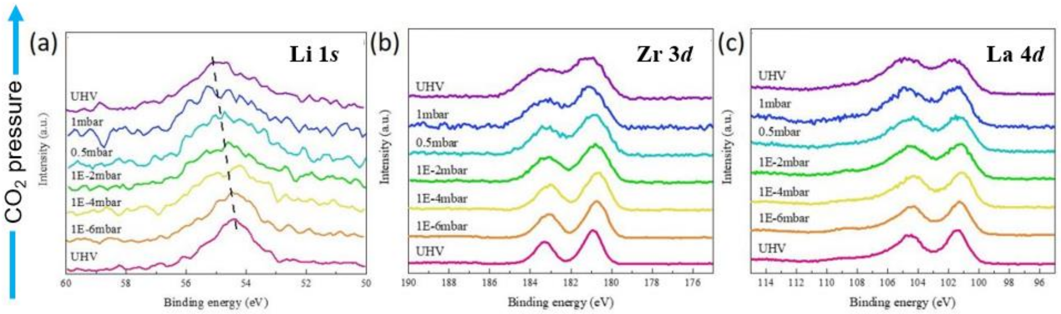

Supplementary Fig. 8 | The variation of Li 1*s*, Zr 3*d* and La 4*d* APXPS spectra at increasing CO_2 pressure. The peak position of Li 1*s* shifts to high binding energy indicates the change from LLZO to Li_2CO_3. No obvious changes can be found in Zr 3*d* and La 4*d* spectra. The signals of La and Zr are weaken with increasing CO_2 pressure (signal-to-noise ratio), indicating that lithium carbonate forms on the surface of LLZO.

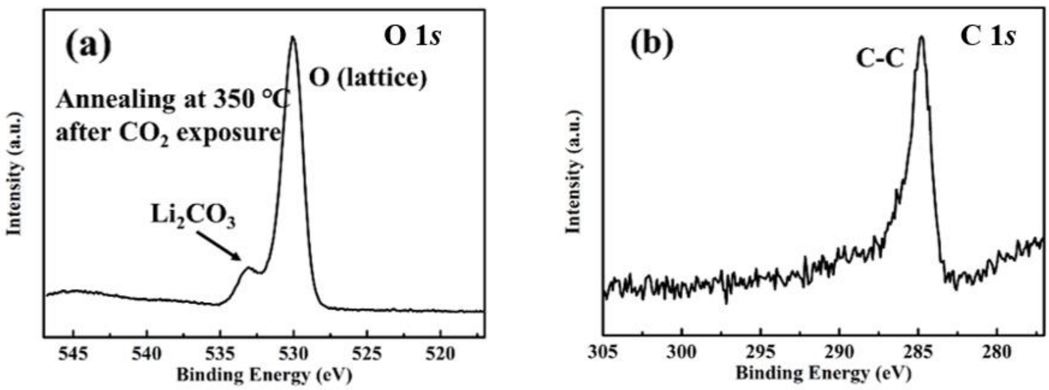

Supplementary Fig. 9 | XPS spectra of LLZO surface when vacuum annealing at 350 °C after CO_2 experiment. a O 1*s* and **b** C 1*s* XPS spectra of LLZO which is annealing at 350 °C after the CO_2 experiment, corresponding to process d in Fig. 2 of the main text. The results indicate that Li_2CO_3 is difficult to decompose at 350 °C if H in the sub-surface of LLZO is completely removed.

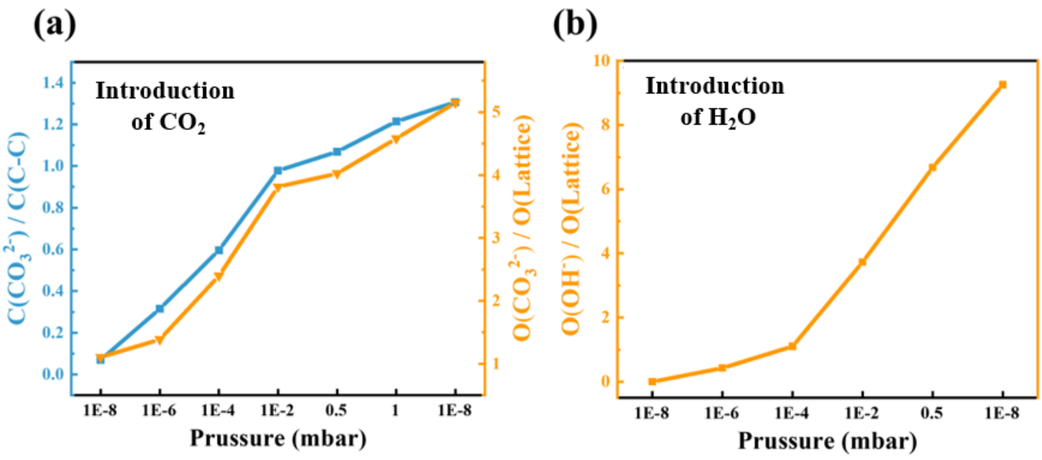

Supplementary Fig. 10 | Evolution of surface species during the introduction of CO_2 and H_2O. a The variation of $C(CO_3^{2-})/C(C\text{-}C)$ and $O(CO_3^{2-})/O(Lattice)$ during the introduction of CO_2. **b** The variation of O(OH-)/O(Lattice) during the introduction of H_2O. The variation trend of the two ratios is very consistent in CO_2 procedure. The ratios of $C(CO_3^{2-})/C(C\text{-}C)$ and $O(CO_3^{2-})/O(Lattice)$ increase quickly at low CO_2 pressure from 1×10^{-8} mbar to 1×10^{-2} mbar and stay stable from 1×10^{-2} to 1 mbar. However, for H_2O, the ratios of $O(OH^-)/O(Lattice)$ change slowly at the pressure below 1×10^{-4} mbar and increase quickly as the increasing of the pressure to 0.5 mbar.

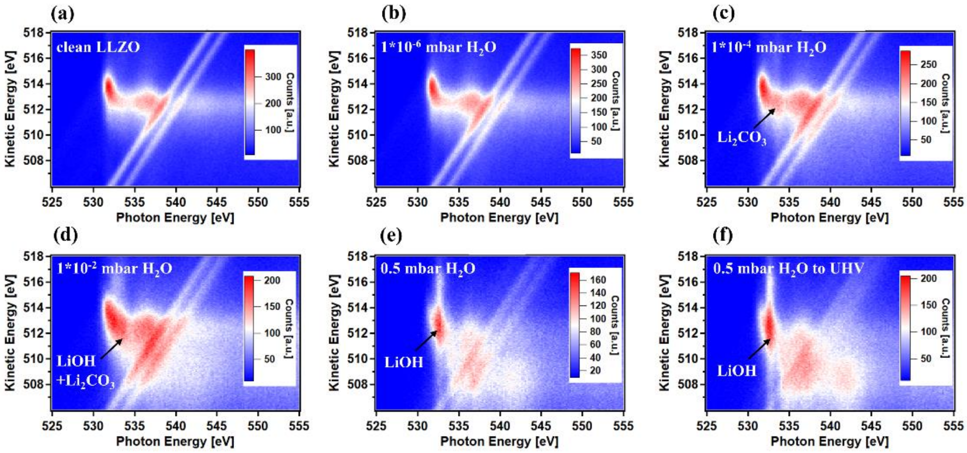

Supplementary Fig.11 | The AP-mRAS spectra of LLZO surface during the introduction of H_2O. Li_2CO_3 can be observed at $1x10^{-4}$ mbar H_2O due to the gas path can only be cleaned to 1×10^{-7} mbar and there is a small amount of residual CO_2. The results also indicate that the reaction of $Li_{6.5}La_3Zr_{1.5}Ta_{0.5}O_{12}$ + $xCO_2 \rightarrow$ $Li_{6.5-2x}La_3Zr_{1.5}Ta_{0.5}O_{12-x}$ + xLi_2CO_3 at clean LLZO surface may be a thermodynamically favorable route compared to the reaction of LLZO with H_2O. When the pressure increases to 0.5 mbar, the signal of LiOH almost completely covers that of LLZO.

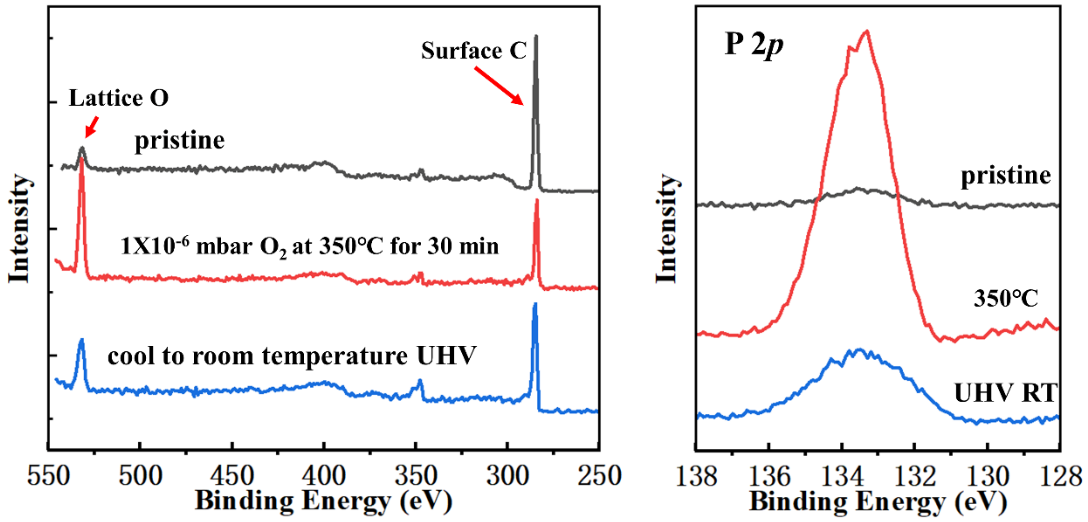

Supplementary Fig.12 | Evolution of surface species on LAGP during annealing and cooling processes. It can be seen that the surface C of the sample at 350 ℃ significantly decreases, while the signals of O and P are significantly enhanced. However, when the sample was cooled to room temperature, the signal of C increased significantly while the signal of O and P decreased. No such carbonization phenomenon is observed on the surface of LLZO.

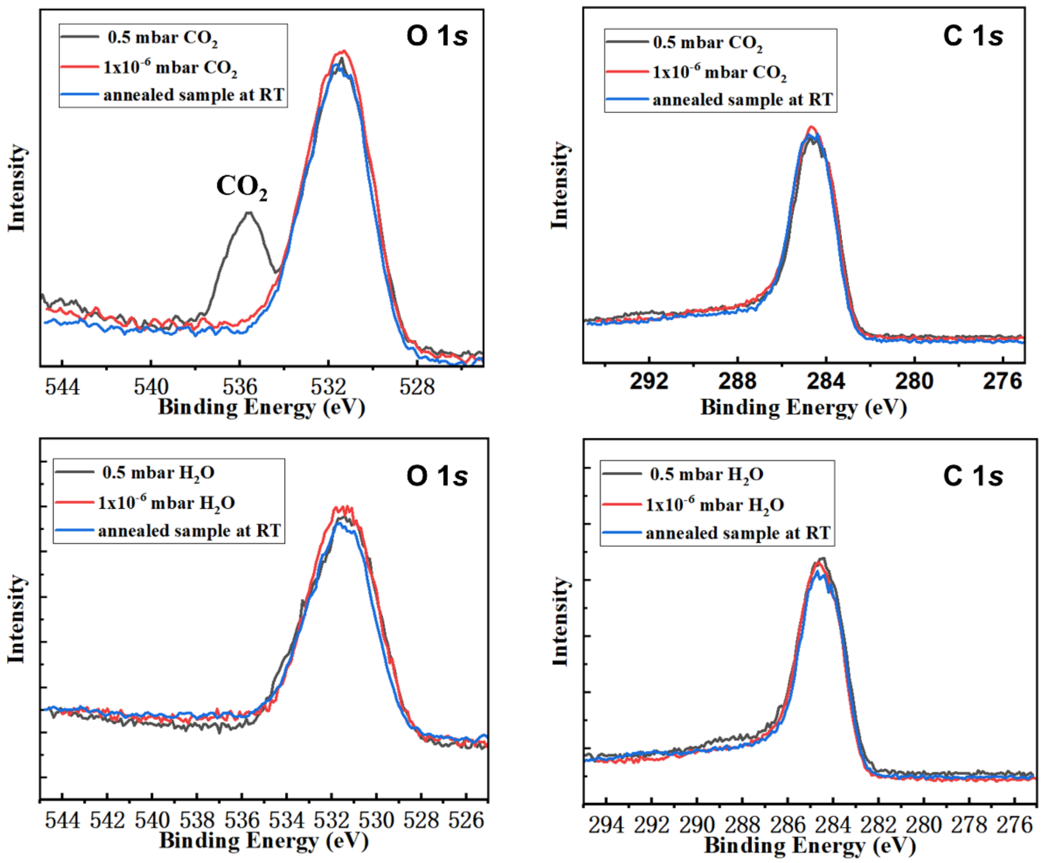

Supplementary Fig.13 | *In situ* ambient pressure experiment of LAGP sample with CO_2 and H_2O. No changes can be found in O 1*s* and C 1*s* spectra. Thus, LAGP cannot react with CO_2 and H_2O even at a high pressure of 0.5 mbar.

	**Area of Li_2CO_3**	**Area of LiOH**	**Area Li_2CO_3/LiOH**	**Molar ratio**
**UHV**	40207.7	2369.6	16.96	5.65
**0.5 mbar H_2O+0.5 mbar CO_2**	3981.7	363	10.97	3.66
**UHV**	12856.6	36133.1	0.35	0.12
**0.5 mbar H_2O**	1178.2	28552.1	0.04	0.01
**$1X10^{-2}$ mbar H_2O**	19551.1	39485.7	0.50	0.17
**$1X10^{-4}$ mbar H_2O**	30297.5	12850.1	2.36	0.78
**$1X10^{-6}$ mbar H_2O**	32278.6	6112.3	5.28	1.76
**UHV**	33434.8	0	--	--

Supplementary Table 1 | Changes of Li_2CO_3/LiOH on the surface of LLZO during the H_2O reaction. The processes are corresponding to Fig. 6 in the main text. Li_2CO_3 rapidly increases under low pressure because our gas path can only be cleaned up to 1×10^{-7} mbar, and there still contains a small amount of CO_2 in the gas path. LiOH increases continuously, indicating that there is almost no LiOH present on the UHV sample. After the introduction of H_2O+CO_2, LiOH almost changes to Li_2CO_3.
